# Views and experiences on writing certificates for assisted dying: interviews with Swedish physicians

**DOI:** 10.3389/fpsyt.2025.1580657

**Published:** 2025-06-18

**Authors:** Filip Jonsson, Manne Sjöstrand, Ulrik Kihlbom

**Affiliations:** ^1^ Department of Learning, Informatics, Management and Ethics, Karolinska Institutet (KI), Stockholm, Sweden; ^2^ Center for Psychiatry Research, Department of Clinical Neuroscience, Karolinska Institutet (KI), Stockholm, Sweden; ^3^ Centre for Healthcare Ethics, Department of Learning, Informatics, Management and Ethics, Karolinska Institutet (KI), Stockholm, Sweden

**Keywords:** physician-assisted suicide, assisted dying, end-of-life, professional ethics, medical ethics, Sweden, semi-structured interviews

## Abstract

**Intro:**

The only legal option for Swedish patients who desire assisted dying (AD) is to travel to Switzerland. To access AD there, patients need medical certificates from their physicians. However, Swedish healthcare law and professional ethical guidelines lack clear directives on how physicians should handle such requests, which may place physicians in perceived ethical and professional dilemmas. How physicians reason about their professional involvement in writing such certificates has previously not been studied in a Swedish context. The aim of this study was to describe and explore physicians’ opinions and reasoning when confronted with requests for AD or requests to enable AD in Switzerland.

**Material and methods:**

12 semi-structured interviews with physicians from different specialties (oncology, neurology, palliative care, psychiatry, general practice, internal medicine) were conducted, transcribed, and analyzed using thematic analysis.

**Results:**

Participants felt it was important to address the reasons why patients wanted to pursue AD, including addressing fears, optimizing care, and existential aspects. Participants felt that they should write certificates to enable AD, citing different reasons. Simultaneously, many participants argued that performing AD in Sweden should not be part of their professional role. Some participants were more positively inclined but were still concerned with perceived obstacles.

**Conclusion:**

Participants were concerned with the underlying reasons for patients pursuing AD, hoping to address them. Interestingly, although many of the participants expressed skepticism towards AD and its legalization in Sweden, they still supported writing a medical certificate enabling AD in Switzerland.

## 1 Introduction

Assisted dying, AD, has long been a controversial topic in Sweden. Recently, the topic has resurfaced around the case of a retired Swedish physician who lost his medical license when he was involved in the death of an individual with amyotrophic lateral sclerosis (ALS) (1, 2). In the current divide between patients’ wishes for assisted dying and Swedish physicians’ current practices, complex ethical dilemmas arise.

AD is when a patient dies by the voluntary, informed, and competent decision to self-administer a lethal drug, which has been prescribed to the patient by a physician for that specific purpose (3, 4). This is differentiated from voluntary euthanasia, where physicians both prescribe and administer the lethal drug after a competent patient’s voluntary request (3, 4). It should be noted that controversy exists around which term is most appropriate and the effects of using different terminology (5). Prior studies and discussions on this topic in Sweden (6–9) have used the terms physician-assisted suicide and assisted dying. AD is legal and practiced in some form in several jurisdictions around the world (4), but not in Sweden (10, 11). Moreover, AD is explicitly prohibited by the professional ethical guidelines published by the Swedish Medical Association (12). Because AD is not practiced in Sweden, Swedish patients’ only accessible option for AD is to travel to Switzerland where it may be accessible from independent organizations, given certain conditions. To establish that these conditions are met, patients must provide a medical certificate to attest to diagnosis, prognosis, and treatment (13). The patient’s decision to pursue AD must be competent and voluntary, but it is not explicitly stated if it has to be included in the certificate (13–15).

In Sweden, physicians have an obligation to, at the request of the patient, provide patients with a written health report containing relevant information about their diagnosis and treatment (16). However, the duty to write a certificate may, in the context of Swiss AD, be perceived to come into conflict with the duty to provide quality and competent care as specified by professional guidelines and Swedish healthcare authorities (9). However, the Swedish National Board of Health and Welfare has not issued any directives on the issue of writing medical certificates for AD.

There are no available data concerning Swedish patients who travel to Switzerland for AD. A review (17) showed that cancer was the most common underlying diagnosis for patients receiving AD in the USA, the Netherlands, and Belgium. Among non-Swiss citizens being granted AD in Switzerland, neurological disease is the most common underlying diagnosis (47%), followed by cancer (37%) (18). Therefore, it can be hypothesized that neurologists and oncologists are the most likely specialists involved in Swedish cases of Swiss AD.

It is likely that physicians who are approached by patients seeking AD may perceive requests for a letter that de facto enables AD as ethically and/or legally problematic. However, little is known about how Swedish physicians reason ethically or what they perceive as important when confronted with AD requests. When surveyed in 2020 (6), results suggested 47% of physicians were positive towards legalizing AD with notable shifts in specialties compared to 2007. Among psychiatrists, a majority accepted AD 2020, significantly higher than in 2007, when only a minority within those specialties accepted AD. In oncology, acceptance rose from 26% to 46%. A different survey (7), conducted in 2022, has suggested a strong correlation between experience with dying patients and a negative stance towards AD. The same study also concluded that 41% of physicians accept AD (7), which aligns with the 2020 survey (6).

Thus, while AD is neither legal nor accepted by Swedish ethical professional guidelines, requests for AD involve Swedish physicians. However, there is no previous research on how Swedish physicians deal with such requests and or how they reason when approached by patients seeking AD. More knowledge is required to better understand the potential ethical dilemmas physicians face and how these complex and challenging situations can be approached in Sweden and in other countries. Our study aimed to explore physicians’ opinions and reasoning when confronted with patients’ requests for AD or requests to facilitate AD in Switzerland.

## 2 Materials and methods

### 2.1 Participant recruitment and data collection

A combination of purposive and snowball sampling was used to recruit a diverse sample of participants from different specialties who regularly work with palliative patients. Using existing professional networks, clinics that were considered to have physicians with relevant experiences were contacted. Once a contact with a physician had been established, they aided us in recruiting additional participants. The participants were all licensed physicians with specialization and experience in the relevant patient groups. We aimed for diversity in in age, gender, and professional background. All participants were practicing in the greater Stockholm area at the time of the interview.

**Figure 1:**
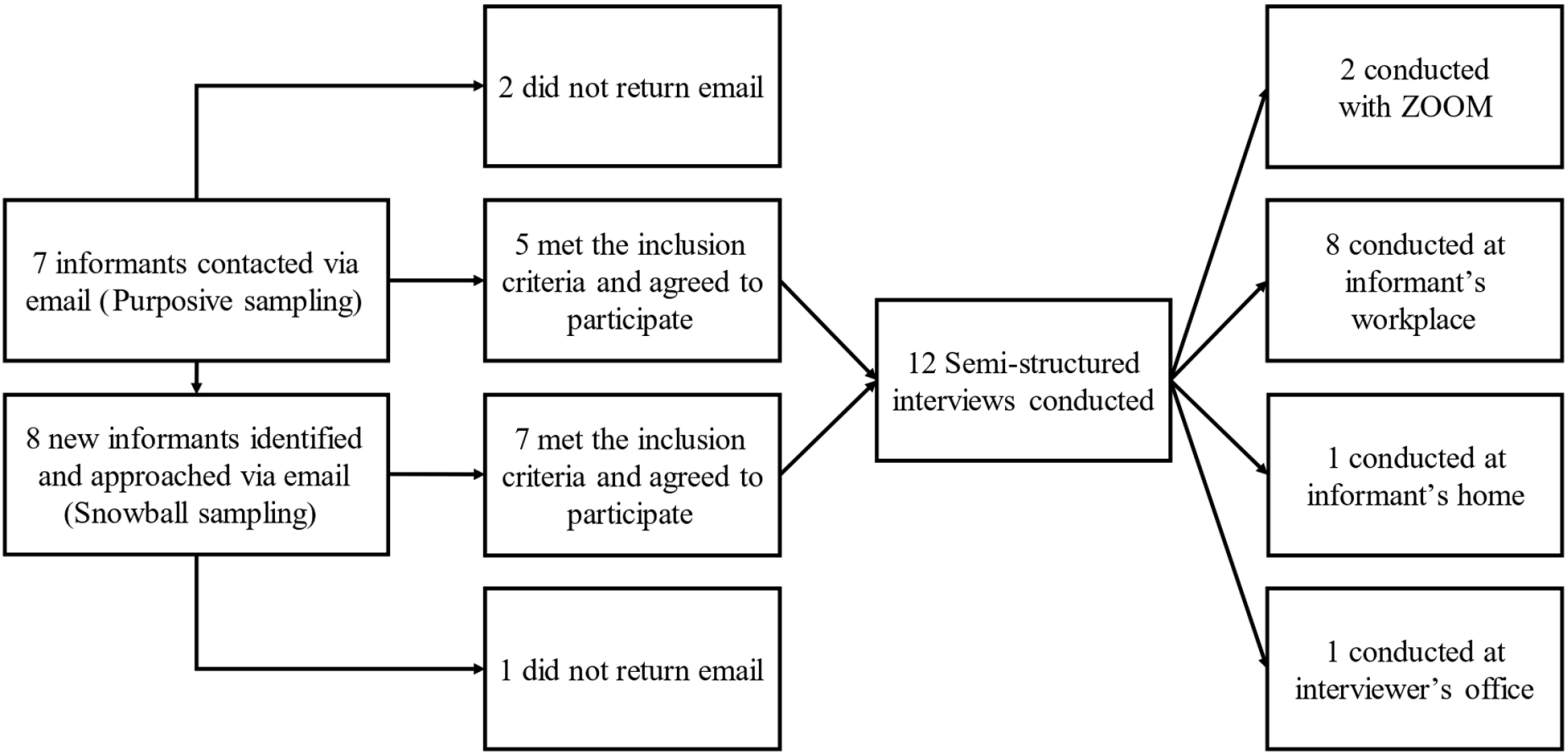
Flow chart showing sampling and setting of interviews.

Potential participants were sent an email with information about the study and participation. Interview date and setting were scheduled according to the participants’ wishes. Written and oral consent were obtained before the start of the interview. The recruitment was done in two instances. First, in March of 2023, participants were recruited both directly and after they had been recommended by a participant. Secondly, participants were recruited during late spring and summer of 2023. An overview of recruitment and interview settings can be seen in **Figure 1**.

The number of participants needed was assessed continuously to achieve information power, as described by Malterud et al. (19). It was easy to recruit participants with specific experience of AD requests and the relevant patient group. The quality of the dialogue between participants (experienced physicians) and interviewer (4^th^ year medical student) was assessed as strong, with interviews lasting between 25 and 62 minutes. This approach supported a limited sample size, however, given the relatively broad research question and limited use of theoretical perspectives, it was finally assessed that 12 participants were necessary for adequate information power.

Semi-structured interviews were used for collection of data. The interview guide consisted of five open-ended topics of discussion (prior experience, patient-physician interaction, important aspects of handling AD cases, whether they would write certificates, and their own opinions of AD). A patient case was included in the interview guide to be used in case of no prior experience when asked the first question. The case concerned a patient with a life-threatening disease, who had previously been assessed by physicians as possessing sound judgement, and who was now requesting the certificates required by Dignitas for AD (13). The participants were specialized in palliative care and/or neurology, oncology, psychiatry, internal medicine, and general practice. See **Table 1** for demographic data. Their prior experience with AD requests ranged from having written the necessary medical certificates multiple times to having only general familiarity of AD requests. One participant had no prior experience with AD requests despite working with relevant patient groups.

**Table 1:** Demographic data of interviewed physicians.

Gender	4 men, 8 women
Age	40–68 years
Experience	6–30 years

### 2.2 Data analysis

We used thematic analysis as described by Braun and Clarke (20). Following the proposed six phases of thematic analysis, FJ first transcribed the interviews and engaged in “repeated reading” of the data (phase 1). Extracts were then coded manually for their semantic content (phase 2) and inductively organized into potential themes by FJ (phase 3). This was done to develop a rich description of the data set. Potential themes were then revised by MS and UK (phase 4). After discussion among authors, the final themes and subthemes were defined and named (phase 5), and a final report was drafted and reviewed by authors (phase 6). FJ translated condensed data extracts, citations used for the report, and codes from Swedish to English.

The analysis conducted used a realist approach. A realist approach assumes that the analysis of the language participants use (our interview data) captures “individual subjective perceptions of real true phenomena” (21). As the goal was to explore perspectives grounded in clinical experience and moral reasoning, we consider this approach is appropriate. This is consistent with Sandelowski’s argument that “a factist approach to interview data” is appropriate for thematic analysis in qualitative descriptive studies (22).

### 2.3 Ethical permit

Ethical permit was granted by the Swedish Ethical Review Authority, record number 2022-07105-01.

## 3 Findings

The analysis resulted in three themes, with additional descriptive subthemes. The three themes were “Addressing reasons to pursue AD”, “Reasons to provide the requested certificates” and “Obstacles for providing Swedish AD”.

### 3.1 Addressing reasons to pursue AD

**Figure 2:**
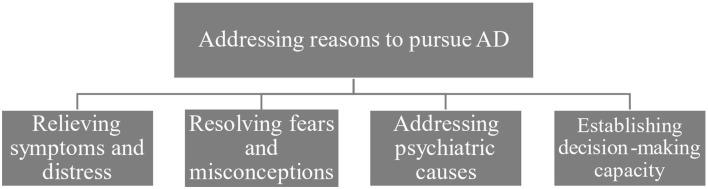
The theme “Addressing reasons to pursue AD” branching out into descriptive subthemes.

All participants addressed the importance of assessing patients’ needs and medical status, emphasizing this as a key aspect of the assessment. The motivation for doing this was to address the underlying cause, as it was common to assume that a request for AD could be connected to some kind of underlying suffering. An overview of the theme and subthemes can be seen in **Figure 2**.

#### 3.1.1 Relieving symptoms and distress

How to optimize patient care was seen as an important assessment. All participants that a request for AD could mean that the patient was suffering from untreated symptoms, or not receiving adequate care, with 11 out of 12 participants viewing it as their responsibility to assist the patient in exploring all available options. For instance, one participant revisited the topic when a patient unexpectedly requested the necessary medical certificates, to ensure that the patient had understood the alternatives.

“We had already talked about this topic, alleviation of symptoms and the possibility of an ambulatory infusion pump and the possibility of palliative sedation … but what felt important to me in that situation was that she, you know, understood what care and support she could get here, as an alternative.” Participant 9

#### 3.1.2 Resolving fears and misconceptions

All participants highlighted the importance of addressing fears and misconceptions, noting that fears and misconceptions about dying were common in patients expressing a wish for AD or necessary certificates.

“As I said earlier, there are many, who express thoughts about not wanting to live to the end, that want to end it before. In those cases, it is often about, in my opinion, the patient imagining an agonizing end without dignity.” Participant 7

#### 3.1.3 Addressing psychiatric causes

The need for psychiatric evaluations was a common topic and was brought up by 11 participants. This included both addressing possible underlying psychiatric disorders and trying to establish that the patient’s desire for AD was not due to psychiatric causes.

“Then we need a psychiatrist who can attest [ … ] that yes, the depression is gone, but thoughts about dying remain. Primarily existential thoughts about dying, in comparison to depressive thoughts.” Participant 6

“Not all suffering is psychiatric in nature. There are psychiatrists who believe that all suicides are due to mental illness, and that is nonsense. That is my opinion, I cannot understand how they can believe that.” Participant 7

#### 3.1.4 Establishing decision-making capacity

Almost half of the participants (six) stressed the importance of assessing the patient’s capacity to make informed decisions when a patient presented with a request for AD or necessary medical certificates. Specifically, dementia was brought up as a factor that could affect patients’ capacity. It was also stressed that the desire for AD or necessary certificates should be consistent over time and that time was also needed to assess the patient’s capacity.

“You must be able to reason, receive the information that I present as their physician, and still maintain the decision to proceed while understanding the consequences.” Participant 6

“So, that is another aspect of this, that you have time. You need time to see, also when it comes to the question of whether the patient has capacity, you need time. You want to have the possibility to check if this really is a persistent desire. Does this hold if things change?” Participant 7

### 3.2 Reasons to provide the requested certificates

**Figure 3:**
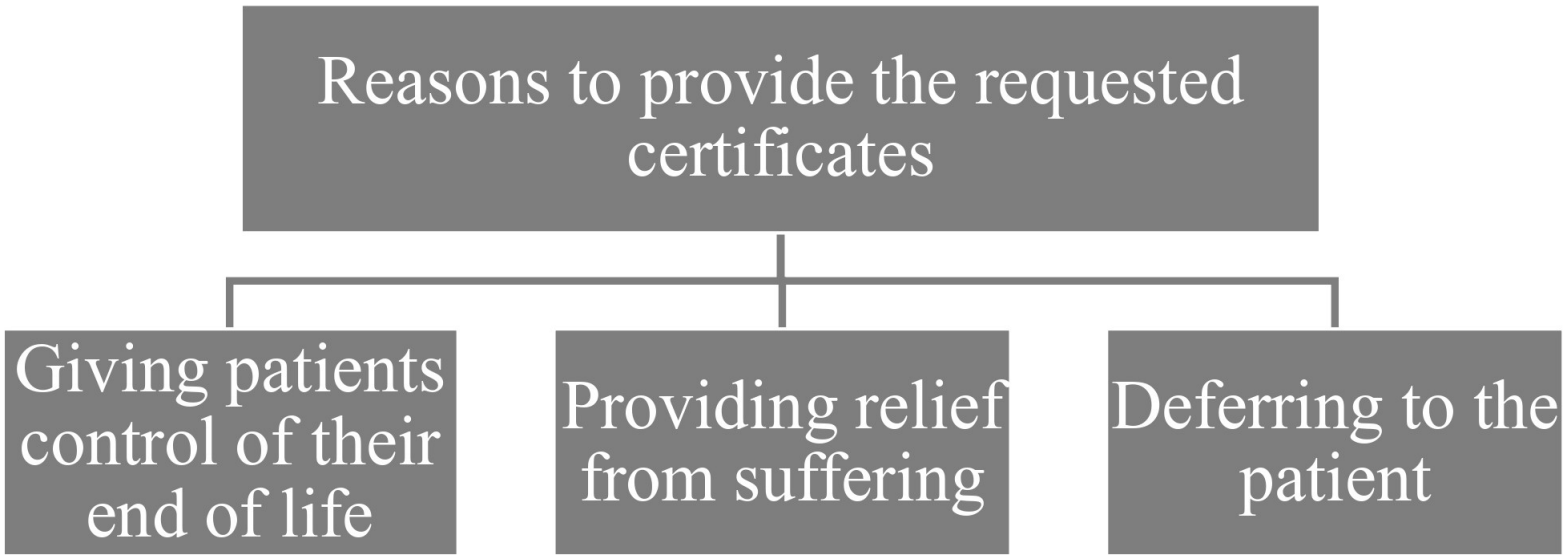
The theme “Reasons to provide the requested certificates” branching out into descriptive subthemes.

The participants were willing to write the necessary certificates for AD, with 10 of 12 participants explicitly stating that they would have or had written certificates. This does not suggest that they saw this as a straightforward decision. Rather, the interviews explore considerations of various factors, focusing on giving patients control and providing relief from suffering, where the focus was on the notion of existential suffering, which we will return to in the discussion. Ultimately, eight of the participants wanted to defer the decision to the patient, possibly while remaining neutral on the question of whether to endorse the patient’s desire for AD. An overview of the theme and subthemes can be seen in **Figure 3**.

#### 3.2.1 Giving patients control of their end-of-life

Providing medical certificates was seen as important to strengthen the patient’s autonomy and control over matters at the end of life. Participants explained that one of the important reasons patients requested medical certificates from them was that it gave the patients more control of their end-of-life compared to what they could otherwise offer.

“Then you decide the time. That is what is important: on Monday, three weeks from now, then my life will end. Then you are in control, whereas what we offer, that there will be aid somewhere near the end of life … it is not enough for everyone.” Participant 6

“Yes, it is difficult. At the same time, absolutely … even if I may contradict myself … it is clear that if you have a slow progressive disease, you should have the right to decide about your life.” Participant 5

#### 3.2.2 Providing relief from suffering

The topic of suffering was frequently raised by the participants. Here, a distinction was sometimes made between suffering due to painful symptoms, which could potentially be relieved by medical measures, and suffering that was existential and potentially beyond what could be alleviated through care and treatment. Three participants described it as common in their patient group without directly associating it with AD, while six participants linked it to requests for AD and the necessary certificates. According to the participants, it was not a reason to deny the request. Rather, it was seen as a reasonable explanation for the patient’s pursuit of AD. One participant discussed a case where a patient requested palliative sedation.

“It is severe symptoms and perhaps primarily a severe existential suffering. That you cannot bear your situation. It does not have to be severe physical symptoms.” Participant 8

Another participant reasoned that, perhaps, the reason for their patient requesting certificates for AD could have been because it was very difficult on an existential level to await death and the uncertainty of how that time would be.

“It was probably existentially very difficult to wait for that [death], how, how will this be and what help will I get, and will it be dignified, in a way.” Participant 9

#### 3.2.3 Deferring to the patient

A common reasoning was that, regardless of the participant’s stance towards the request of AD, it was still reasonable to defer to the patient. After all, many argued, the certificate contains medical information the patient has the right to access, and it is up to the patient to use the information as they see fit.

“I have no problem with it. It is the patient’s property, so to speak. So that I can … It would be weird if I said that no, you will not get one.” Participant 12

Four participants added that they did not address the certificate to an assisted dying organization or specify the purpose, also deferring to the patient to use their information as they see fit.

“It wasn’t specified to whom it was addressed to, so to speak, I didn’t address it to someone.” -Participant 9

### 3.3 Obstacles to providing Swedish AD

**Figure 4:**
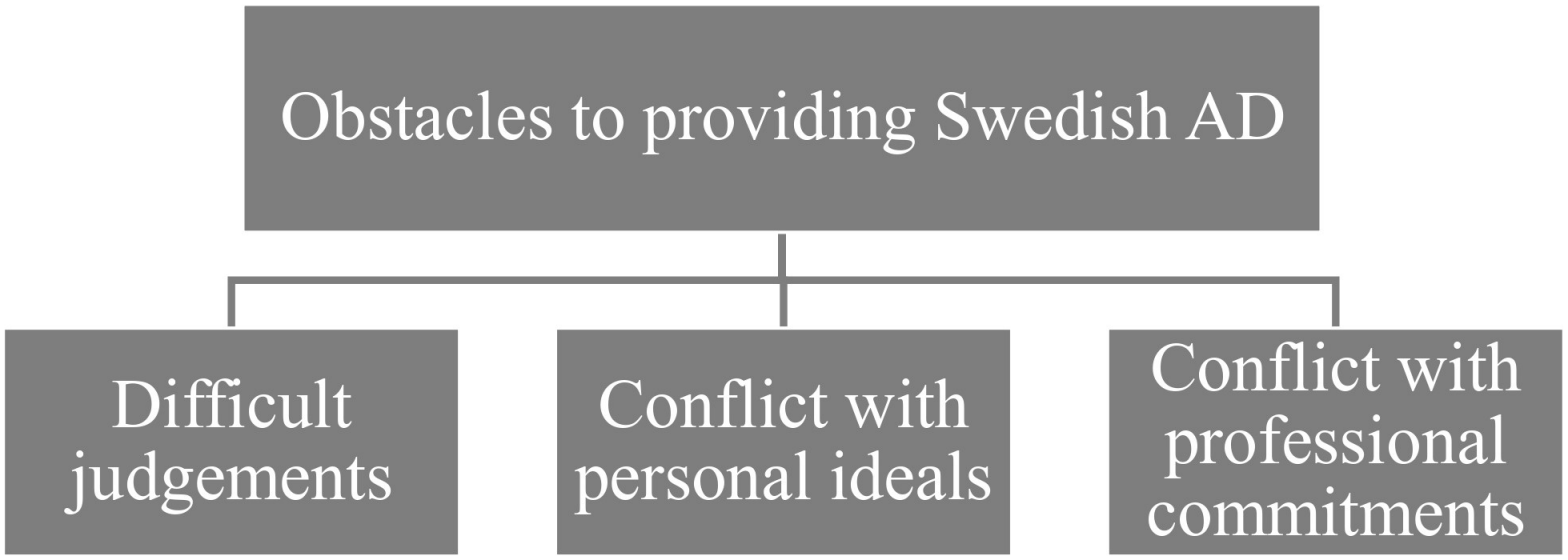
The theme "Obstacles to providing Swedish AD" branching out into descriptive subthemes.

Participants held opposing views on whether AD should become legalized in Sweden or not, but all participants detailed obstacles to providing AD. Six participants argued that obstacles needed to be addressed to provide AD in Sweden, possibly reflecting a positive or neutral attitude, while six participants concluded that these obstacles meant that AD should not be legalized in Sweden. An overview of the theme can be seen in **Figure 4**.

#### 3.3.1 Difficult judgements

When discussing the hypothetical responsibility of providing AD in a Swedish context, a recurring topic was the many difficult judgments that participants associated with the request. These included which diagnoses should be accepted as reasons to pursue AD or the risk that family members were unduly influencing the patient’s wish for AD. When participants indicated that they would accept AD, they stipulated requirements about information and capacity, including a thorough understanding of the patient, possibly including several assessments over time, to consider providing AD.

“Because then I as a physician have a deep understanding of this person’s understanding of their situation and life. An assessment has been made that shows that they aren’t mentally ill. They have received all information about how their dying process would progress … and still they have chosen this … Then I would think it is ok.” Participant 11

#### 3.3.2 Conflict with personal ideals

It was evident that five participants could not envision themselves providing AD due to personal barriers or stances even if they take a non-directive stance.

“Not that I think it is wrong. I find it difficult to say that, because I’m not the person that is in that situation. I’m not directly against that people travel for assisted dying. I just know that I wouldn’t be able to work at such a place.” Participant 1

#### 3.3.3 Conflict with professional commitments

Participants also argued that AD would clash with professional commitments, such as ethics or the role of providing palliative care. Five participants concluded that, if legalized, assisted dying should be handled by a different profession altogether.

“Then you would have to train someone else, a ‘death patrol’, who are to give this injection, because as a physician I think it completely unethical to inject a patient with a lethal medication.” Participant 2

## 4 Discussion

When seeking to understand the underlying reason for a patient pursuing AD, participants focused on potentially treatable or reversible factors. Additionally, they aimed to identify and resolve faulty or mistaken reasoning behind the desire to die. However, when factors appeared to extend beyond the scope of treatable symptoms, participants were more accepting of AD requests. Here, some participants brought up the notion of existential suffering. No unified definition of existential suffering was provided, but it was characterized as stemming from the totality of the patient’s situation and suffering and related to the uncertainty of when death will occur. No consensus definition exists, and various clinical manifestations may be considered existential suffering (23). Still, the concept seems to play a role in patients’ desires to hasten death (24), and it was used by participants in a Swiss study (25) to delineate symptoms that they perceived beyond the scope of medicine to alleviate. It was also alluded to in reasoning about the difference between AD and psychiatric suicide. Here, participants saw a morally relevant difference between an existential desire to die and suicidal ideation that was caused by mental illness. Ruling out the latter was considered necessary for writing a certificate enabling AD. The possible acceptability of AD and suicides that are not caused by psychiatric disorders is also explored in a newly published Swedish study on psychiatrists’ views and experiences of so-called rational suicides (26).

Most of the participants in this study expressed a willingness to write the necessary medical certificates for AD. This is somewhat surprising, considering that AD is controversial in the context of Swedish healthcare. A previous survey of Swedish physicians showed that half of the respondents were willing to write the necessary medical certificates (7). As noted, there are possible legal and/or professional ethical questions, but there are no official guidelines to rely on. It is, however, likely that the willingness to write certificates for AD varies between different specialties and personal experiences. For this study, we purposely recruited physicians with relevant experience where requests for AD are likely to occur. Personal experiences may affect general moral judgments and attitudes both to AD in general and writing certificates enabling AD.

Our study described that some of the participants would be against providing AD in a hypothetical Swedish setting due to conflicts with personal ideals and conflicts with professional commitments. This finding is not surprising, given that research in jurisdictions where AD is practiced has shown that physicians may be reluctant to provide AD or euthanasia due to personal or professional values (27, 28). In Switzerland, a survey study showed that 22% of physicians fundamentally oppose AD, but it did not explore the underlying reasoning (29). A Swiss interview study found that religious beliefs could contribute to participants refusing an active role in providing AD (27), but this was not provided as a reason by participants in our study. It is interesting to note that although some participants expressed skepticism towards legalizing AD in Sweden, even strongly rejecting direct professional involvement in hypothetical Swedish AD, they were still willing to write the necessary certificates for AD in Switzerland.

It may seem counterintuitive that providing certificates to enable AD is perceived differently than directly assisting in dying. A relevant difference could be the burdens connected to providing AD. To compare, a 2019 interview study with physicians who provided AD during the first years of its implementation in the Australian state Victoria reports a lack of emotional support and that participants would limit their involvement in cases that did not align with their personal values (28). In a Dutch survey, 49.6% of physicians reported feeling “burdensome” after their most recent granted request for euthanasia (30). The physicians in the present study did not directly report emotional or moral distress related to writing the certificates. However, writing certificates does not require physicians to finally determine whether a patient’s request should be accepted, which may be seen as less emotionally or ethically challenging.

However, a possible tension is reflected in the practice of writing the certificates. As noted, participants may be willing to issue certificates for AD while avoiding a clear statement of purpose. This could be seen as reflecting an underlying legal and ethical uncertainty. It may also be a way for the physicians to resolve a perceived conflict between AD, which many participants were reluctant to endorse, and respecting patient autonomy. Nevertheless, such a practice is not in line with the general recommendations from the Swedish National Board of Health and Welfare which specify that both the purpose and addressee should be clearly stated in a medical certificate (16).

Participants claimed that providing AD in Sweden would infer difficult judgements related to, but not limited to, how to make sure the patient is not unduly influenced by family members, which international literature also suggests is part of the overall assessment. A Dutch interview study, focused on perceived complex cases, has previously shown that relatives are an important part of the AD/euthanasia process that influences the clinicians’ decisions (31). A Dutch survey also showed that 31% of physicians, when they had refused a request for euthanasia, felt pressured to grant the request by relatives (30). When elaborating on their own hypothetical involvement in AD cases, participants stipulated that several assessments of time would be necessary to correctly assess a request for AD. Previously, a survey study in Sweden has shown that a majority of physicians believe that a specialized clinic would be the most appropriate setting for providing AD (7).

For Swedish healthcare policy, the uncertainty of present practices needs to be addressed. It is likely that patients with AD requests receive different responses from their treating physicians regarding whether certificates enabling AD may be issued. The lack of clarity can result in inconsistent practices between different healthcare services and possibly also within the same hospital. Such cases would come into conflict with the fundamental principle providing “equal access for equal need” that is a basis for the Swedish health care system (10). The uncertainty and potential conflict between obligations may also result in moral distress, but this was not brought up by the participants in this study.

### 4.1 Strengths and limitations

By including different specialties and using a broad approach to the subject, a rich dataset was acquired. It enabled the exploration of physicians’ opinions and experiences, adding novel data and insights. Previous surveys have only been able to ask yes and no questions, and the findings of this study bring needed nuance to previous quantitative studies. However, the limitations of this study must also be recognized. The participants were recruited with the purpose of shedding light on the question of AD requests. Focusing on physicians with experience of palliative patients and AD requests was a deliberate choice to gather informed perspectives, but the selection does not represent Swedish physicians in general. Given that access to palliative care differs in Sweden, with the Stockholm greater area having higher access to palliative care than most other regions in the country (32), it should also be noted that responses from participants might not be representative of physicians in rural areas or areas with less access to palliative care.

## 5 Concluding remarks

For future research, it would be interesting to test if our current finding, that participants were willing to write the certificates necessary for AD in Switzerland, is true for other patient categories, such as patients with disability or chronic pain. Given the rarity of patients pursuing these certificates, a survey study asking physicians if they have been asked to write these certificates and what they perceived to be the main reason behind it would further our understanding of this phenomenon, also allowing for purposive sampling of physicians who have written certificates for a follow-up interview study.

For the participants, it was important to address why a patient would want to pursue AD, but they were, in general, willing to write the necessary certificates enabling AD. In contrast to this willingness, many participants voiced skepticism about the possible introduction of AD in Sweden. Interestingly, the participants do not seem to perceive writing certificates as much of a dilemma, believing it aligns with values such as giving patients control or deferring to the patients. Instead, dilemmas arise in the difficult question of how to reasonably detect faulty or mistaken reasoning for wanting to die, and if AD is legalized in Sweden, it perhaps needs to consider the physicians who are supportive of patients and their requests but oppose providing AD themselves.

## Data Availability

The data consists of transcripts of semi-structured interviews with participants (in Swedish). The transcripts, while they don’t include names of participants, contains enough personal information to potentially reveal the identity of the participant. As such the data cannot be accessed due to confidentiality. Requests to access the datasets should be directed to filip.jonsson@stud.ki.se.
