# Supplementary material for: Views and experiences on writing certificates for assisted dying: interviews with Swedish physicians
Source: Front Psychiatry. 2025 Jun 18;16:1580657. doi: 10.3389/fpsyt.2025.1580657 (PMC12213817; doi:10.3389/fpsyt.2025.1580657)
Supplement: Supplementary file 1 [file DataSheet1.pdf]

Interview Guide

Warm up question (not to be transcribed):

- How long have you worked as a physician and why did you choose the specialty that you chose?

1 Clinical experience – Have you ever met a patient that have requested/ wanted to discuss physician-assisted suicide (PAS)?

- In what context?
- How did you initially act?
- What kind of information do you consider important for making a decision?
- Do you think your actions affected the patient's decision/wish?

1.2 Patient case - What would you do in this case?

- Initial reaction after the patient's request?
- What kind of information do you consider important for making a decision? What information do you believe is currently lacking in the case?
- Who has the responsibility to provide the patient with their request?
- Do you believe that you have any influence over the patient's decision or wish?
- How would you respond to the patient's questions?

2 The conversation regarding PAS/ the certification enabling PAS

- How do you feel about your role as a physician? Does that change in this context?
- What do you want to know from your patient?
- Do you experience any differences compared to other conversations with patients?

4 Support for future decisions

- In what situations do you think physicians need professional support?
- If you could consider working with PAS in the future, what professional support would you need?
- Which professions need to be involved for adequate care?

5 Own opinions

- Of PAS
- Of relevant ethical principles that guide your reasoning
- Do you believe PAS will be legal in the future?
- Do you believe legalization will change your practice?

Intervjuguide

Uppvärmningsfråga (svaret transkriberas ej):

- Hur länge har du arbetat som läkare och hur kommer det sig att du valt den inriktning du har?

1 Klinisk erfarenhet - Har du någonsin träffat en patient som efterfrågat/diskuterat läkarassisterat suicid (LAS)?

- I vilket sammanhang?
- Initialt agerande vid patients begäran?
- Vilket underlag bedömer du som viktigt för att ta ett beslut?
- Upplever du att ditt agerande påverkade patientens beslut/vilja?

1.2 Patientfall* - Vad skulle du göra i detta patientfall?

- Initialt agerande vid patients begäran?
- Vilket underlag bedömer du som viktigt för att ta ett beslut? Vilken information upplever du att du saknar för att ta ett beslut?
- Vem har ansvar för att tillgodose patientens förfrågan?
- Upplever du att du har inflytande över patientens beslut/vilja?
- Vad skulle du själv svara på patientens frågor?

Etikprövningsmyndigheten
2022-07105-01-346403
2022-12-20

* Se patientfall nedan

2 Patientsamtalet om LAS/intyg för möjliggörandet av LAS

- Hur upplever du dig i din roll som läkare? Förändras den i detta sammanhang?
- Vad vill du veta från en patient?
- Upplever du några skillnader gentemot andra patientsamtal?
  Hur lägger du/tycker du att du bör lägga upp dessa samtal?

4 Stöd vid framtida beslut

- I vilka situationer/frågeställningar tror du läkare behöver professionellt stöd?
- Om du skulle tänka dig att jobba med LAS i framtiden, vilket professionellt stöd skulle du vilja ha?
- Vilka professioners kompetens behövs för ett adekvat omhändertagande?

5 Egna åsikter

- Om LAS
- Om relevanta etiska principer som guidar en i egna resonemang
- Tror du att LAS kommer bli lagligt i framtiden?
- Tror du att det kommer förändra din yrkesutövning?
